# Increased H3K9me3 and F-Actin Reorganization in the Rapid Adaptive Response to Hypergravity in Human T Lymphocytes

**DOI:** 10.3390/ijms242417232

**Published:** 2023-12-07

**Authors:** Kendra Wernlé, Cora S. Thiel, Oliver Ullrich

**Affiliations:** 1 Institute of Anatomy, Faculty of Medicine, University of Zurich, Winterthurerstrasse 190, 8057 Zurich, Switzerland; kendra.wernle@uzh.ch; 2 Faculty of Medical Sciences, Private University of the Principality of Liechtenstein (UFL), Dorfstrasse 24, 9495 Triesen, Liechtenstein; 3 Institute of Machine Design, Otto-von-Guericke-University Magdeburg, Universitätsplatz 2, 39106 Magdeburg, Germany; 4 Space Life Sciences Laboratory (SLSL), Kennedy Space Center, 505 Odyssey Way, Exploration Park, Merritt Island, FL 32953, USA; 5 UZH Space Hub, Air Force Center, Air Base Dübendorf, Überlandstrasse 270, 8600 Dubendorf, Switzerland; 6 Department of Industrial Engineering, Ernst-Abbe-Hochschule (EAH) Jena, Carl-Zeiss-Promenade 2, 07745 Jena, Germany; 7 Zurich Center for Integrative Human Physiology (ZIHP), University of Zurich, Winterthurerstrasse 190, 8057 Zurich, Switzerland

**Keywords:** hypergravity, immune system, histone modifications, nuclear architecture

## Abstract

Our study explored the impact of hypergravity on human T cells, which experience additional acceleration forces beyond Earth’s gravity due to various factors, such as pulsatile blood flow, and technology, such as high-performance aircraft flights or spaceflights. We investigated the histone modifications Histone 3 lysine 4 and 9 trimethylation (H3K4me3 and H3K9me3, respectively), as well as the structural and cytoskeletal organization of Jurkat T cells in response to hypergravity. Histone modifications play a crucial role in gene regulation, chromatin organization and DNA repair. In response to hypergravity, we found only minimal changes of H3K4me3 and a rapid increase in H3K9me3, which was sustained for up to 15 min and then returned to control levels after 1 h. Furthermore, rapid changes in F-actin fluorescence were observed within seconds of hypergravity exposure, indicating filament depolymerization and cytoskeletal restructuring, which subsequently recovered after 1 h of hypergravity. Our study demonstrated the rapid, dynamic and adaptive cellular response to hypergravity, particularly in terms of histone modifications and cytoskeletal changes. These responses are likely necessary for maintaining genome stability and structural integrity under hypergravity conditions as they are constantly occurring in the human body during blood cell circulation.

## 1. Introduction

The gravity of Earth (approximately 9.81 m/s^2^) is a fundamental factor affecting life on Earth. However, human cells and tissues experience additional acceleration forces due to various factors, such as pulsatile blood flow, and advanced technology, like aircraft and spaceflight. Fighter pilots can be exposed to high hypergravity levels, up to 6 Gz during training, and even 9 Gz in high-performance aircrafts [1]. Hypergravity is also used occasionally in medical settings to treat conditions like peripheral obstructive arteriopathies, coronary artery disease, lymphedema and the Raynaud phenomenon [2]. Thus, exposure to hypergravity is encountered in various fields of human activity.

Even under normal Earth conditions, cells in the human body, particularly lymphocytes, experience hypergravity due to their circulation dynamics. These cells receive pulsatile accelerations in the range of 5.5 to 15 m/s^2^ while circulating in the bloodstream [3,4,5,6]. This prolonged exposure to accelerations can have various effects on cell behavior. Studies have shown that hypergravity can affect cellular processes and gene expression in various cell types [7,8,9], including lymphocytes, neutrophil granulocytes, melanocytes, fibroblasts, osteoblasts, chondrocytes, myoblasts, cardiomyocytes and mesenchymal stem cells [10,11,12,13,14,15,16,17,18,19]. Notably, rapid changes in gene expression have been observed in response to as little as 20 s of hypergravity in human myelomonocytic U937 cells and in human Jurkat T lymphocytes [20,21], suggesting that the speed and relative difference in gravitational force alteration could be important triggers for cellular responses [22]. Furthermore, experiments have shown that transcriptome changes in Jurkat T lymphocytes exposed to hypergravity during suborbital rocket experiments can be replicated using an experimental centrifuge on the ground at 9× *g* [23]. Our previous research has also observed changes in histone modifications in Jurkat T lymphocytes [24] and cytoskeletal structures in human primary macrophages [25]. It is unknown by which means cells sense gravitational forces, but it is hypothesized that, as a mechanical force, gravity is transduced into the nucleus via the cytoskeleton, potentially changing nuclear structure, chromatin organization and subsequently transcription [26]. Thus, the gravitational force could be transformed into a nuclear response via outside-in mechanotransducive mechanisms [27,28].

Histone modifications are key regulators in gene expression by mediating chromatin states which dictate a gene’s accessibility for transcription [29]. H3K4me3, is a hallmark histone modification for euchromatin [30], while H3K9me3 is bound to heterochromatin and facilitates linkage of chromatin to the nuclear lamina and anchoring to the nuclear membrane [31,32]. Nuclear deformation can induce a transient decrease in H3K9me3 heterochromatin [33] and subsequently an increase in H3K9me3/H3K27me3 heterochromatin [34]. The linker of the nucleoskeleton and cytoskeleton (LINC) is a complex in the nuclear membrane that, as the name suggests, physically links the cytoskeleton to the nuclear lamina within [35]. It is believed that changes in the cytoskeleton due to external forces may induce the LINC complex to transform chromatin states, ultimately leading to changes in gene transcription [27].

We aimed to investigate the cellular mechanisms and dynamics between histone modifications and the cytoskeleton when exposed to hypergravitational states. In this study, we subjected human Jurkat T-lymphocytes to different hypergravity states within a fixed-angle centrifuge. Histone modifications H3K4me3 and H3K9me3 and the nuclear architecture were then analyzed in these samples through immunofluorescence imaging.

## 2. Results

**Figure 1:**
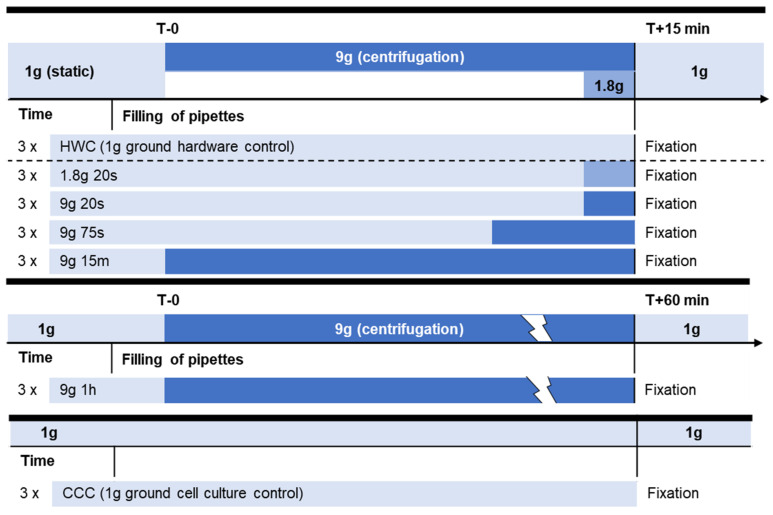
Experiment overview. The experimental conditions from which samples were acquired are displayed. Jurkat T cells were filled into 1 mL pipettes, incubated for 15 min at 37 °C and then rapidly fixed with PFA. In total, there were 7 condition groups, with 3 samples per group. HWC was incubated for 15 min at 1× *g* gravity, the 1.8g, 20s group was incubated for 14 min 40 s at 1× *g* gravity and then exposed to 1.8× *g* for 20 s on a pipette centrifuge before fixation (indicated in light blue), the 9g, 20s group was incubated for 14 min 40 s at 1× *g* gravity and then exposed to 9× *g* for 20 s on a pipette centrifuge before fixation (indicated in dark blue), the 9g, 75s group was exposed for 13 min 45 s at 1× *g* and consequently for 75 s at 9× *g*, and 9g, 15m for 15 min at 9× *g*. Two additional sets contained the group 9g, 1h, which was continuously exposed to 9× *g* for 60 min, and the cell culture control (CCC) which remained in cell culture conditions until fixation.

The primary objective of this study was to investigate the impact of hypergravity on histone modifications and cytoskeletal components in human Jurkat T cells. We conducted a comprehensive analysis of short-term effects, assessing five different centrifugation conditions and incorporating two distinct control groups (Table 1, Figure 1). All experimental sample groups between 20 s and up to 15 min of centrifugation were compared to hardware control samples incubated for 15 min without centrifugation (HWC). Additionally, we included a sample set subjected to 1 h of centrifugation to evaluate any longer-term effects. To distinguish between cellular reactions induced by filling and incubating cells into the hardware, a separate cell culture control group was also incubated without the hardware. The shortest and weakest hypergravity exposure, 1.8× *g* for 20 s, was chosen based on the duration and force of the hypergravity phase on previously performed parabolic flights [20,36]. The 9g, 75s group represented the launch phase of previous ballistic rocket studies [20,36].

### 2.1. Hypergravity Did Not Compromise Cell Viability

For a first impression of cell viability and genome stability between sample groups, we performed a TUNEL (TdT-mediated dUTP-biotin nick end labeling) imaging assay to assess DNA damage following the centrifugation experiment (Supplementary Figure S1). Microscopic examination of TUNEL signals from experimental samples were compared to both positive and negative controls (PC and NC, respectively). Notably, no TUNEL-positive cells were discernable in any of the experimental samples, whereas 100% of the cells in the PC expectedly exhibited TUNEL positivity. Encouragingly, the gravitational forces experienced during centrifugation did not appear to compromise the viability of the cells. These results provide assurance that all samples were suitable for subsequent immunocytochemical staining and analyses.

### 2.2. Hypergravity Only Minimally Affected H3K4me3 Histone Modification

**Figure 2:**
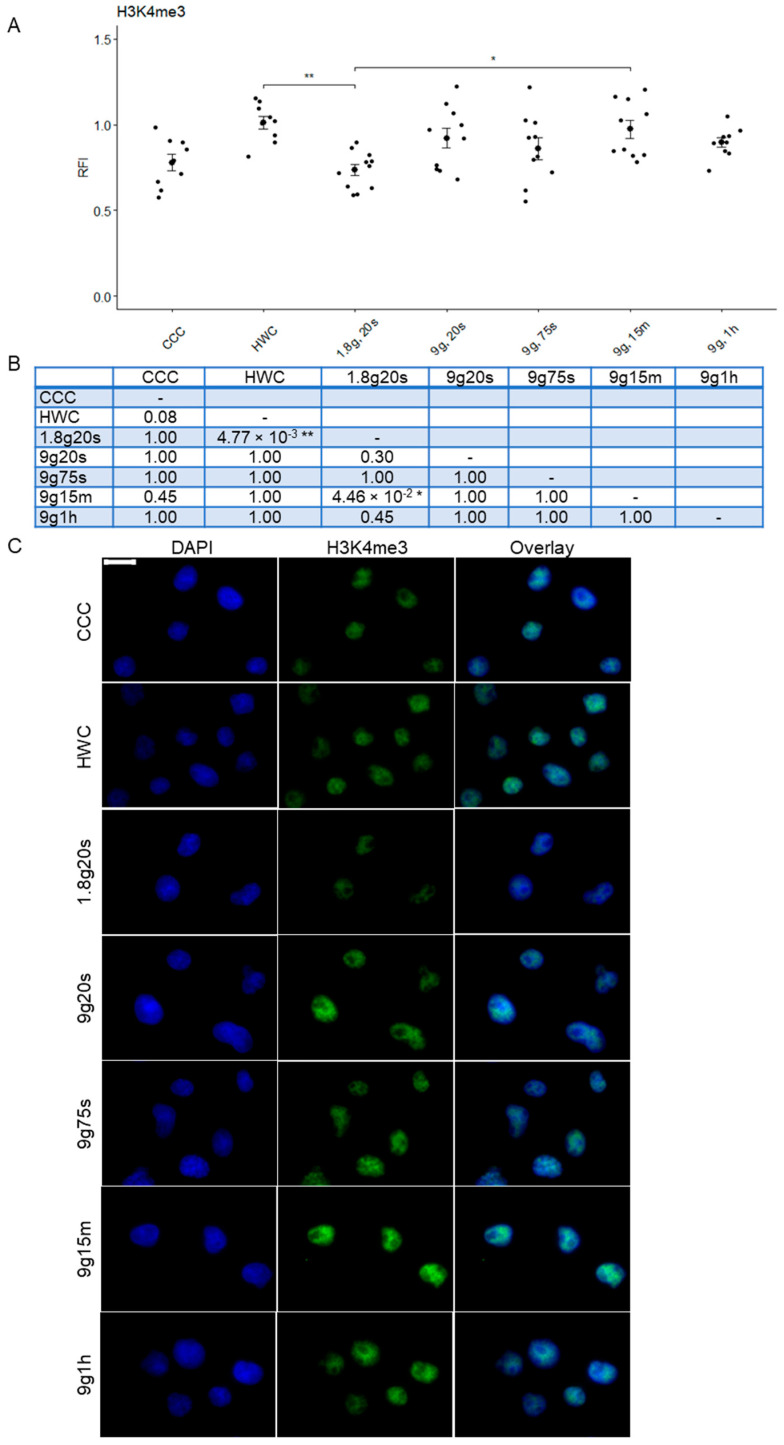
Relative fluorescence intensity (RFI) of histone 3 tri-methylated lysine 4 of Jurkat T cells exposed to different gravity conditions. (**A**) H3K4me3 was visualized with immunofluorescent staining and subsequently analyzed quantitatively. Each data point represents the mean RFI calculated from all cells within a respective image. Error bars indicate SEM. Statistical significance is represented by * *p* < 0.05, ** *p* < 0.01. (**B**) Summary table of *p*-values compared between groups (* *p* < 0.05, ** *p* < 0.01). (**C**) Microscopic images of Jurkat T cells exposed to different gravity conditions. H3K4me3 was stained with Alexa Fluor 488 (green) and nuclear DNA was stained with DAPI (blue). Overlays are also shown. Scale bar 10 μm.

To detect possible changes in the structure of euchromatin upon different gravitational conditions, the Jurkat cells were stained for histone modification H3K4me3 (Figure 2). As shown in Figure 2A, quantification of H3K4me3 staining with relative fluorescent intensity (RFI) revealed minimal significant differences between different hypergravitational states. Our findings revealed that the HWC group (RFI: 1.00 ± 0.0307, n = 755) exhibited nearly 20% higher H3K4me3 fluorescence than the CCC group (RFI: 0.80 ± 0.0411, n = 940). It is important to acknowledge that this discrepancy can be attributed to hardware variations and should be considered during data analysis. Therefore, all results were normalized to the hardware control, with these values set at 100% as a reference point. The 1.8g, 20s group (RFI: 0.74 ± 0.0328, n = 468) displayed the lowest intensity, significantly differing from HWC (*p* = 0.005). In contrast, the 9g, 20s group (RFI: 0.92 ± 0.0589, n = 429) showed an increase in H3K4me3 expression, while the 9g, 75s group (RFI: 0.86 ± 0.0639, n = 546) experienced a decrease. The 9g, 15m group (RFI: 0.97 ± 0.0522, n = 396) demonstrated another RFI increase, with statistical significance compared to the 1.8g, 20s group (*p* = 0.0345). The 9g, 1h group (RFI: 0.89 ± 0.0297, n = 678) exhibited a slight decrease in RFI, falling between those of control samples.

Overall, microscopic immunofluorescent images of nuclei stained with H3K4me3 did not reveal a discernible pattern (Figure 2B). While a slight cyclical pattern of fluorescence intensity was observed among the different time points, with fluorescence showing slight fluctuations between experimental groups, an interesting change was observed between both control groups. Nevertheless, the experimental values consistently remained within the range of control values. This suggests that hypergravity levels and times applied in this work only minimally affect H3K4me3 fluorescence in Jurkat cells, and any variations observed here are well within the expected range.

### 2.3. Dynamic Response of H3K9me3 Histone Modification in Hypergravity Conditions

**Figure 3:**
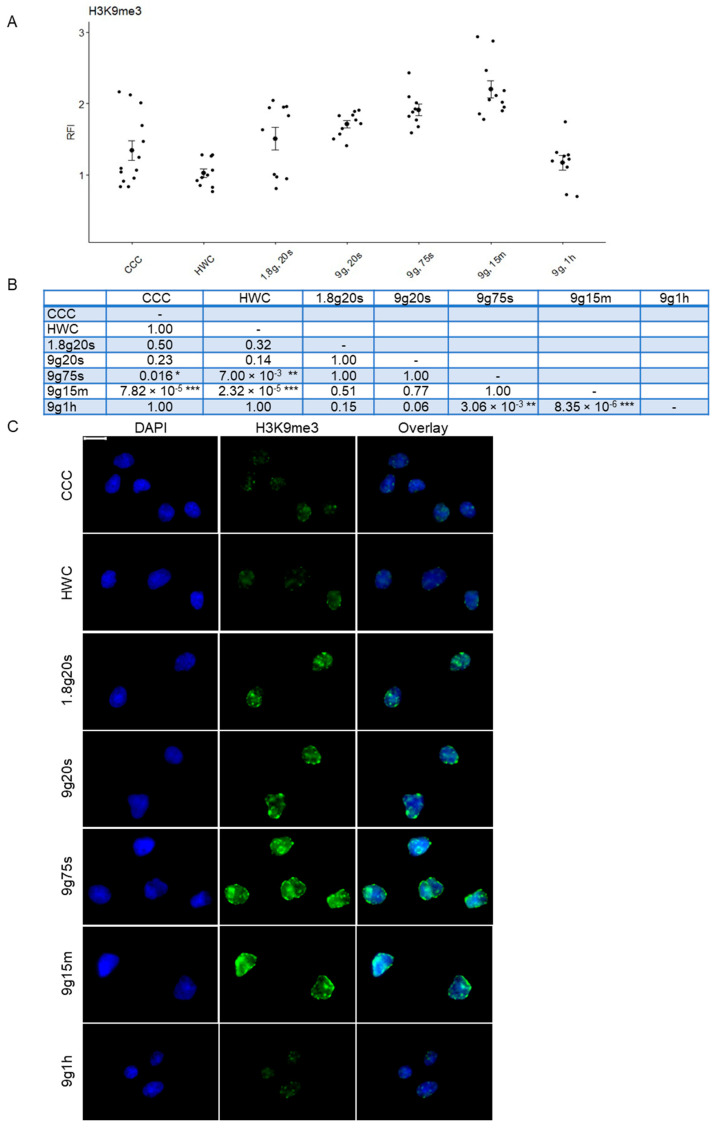
Relative fluorescence intensity (RFI) of histone 3 tri-methylated lysine 9 of Jurkat T cells exposed to different gravity conditions. (**A**) H3K9me3 was visualized with immunofluorescent staining and subsequently analyzed quantitatively. Each data point represents the mean RFI calculated from all cells within a respective image. Error bars indicate SEM. (**B**) Summary table of *p*-values compared between groups (* *p* < 0.05, ** *p* < 0.01, *** *p* < 0.001). (**C**) Microscopic images of Jurkat T cells exposed to different gravity conditions. H3K9me3 was stained with Alexa Fluor 488 (green) and nuclear DNA was stained with DAPI (blue). Overlays are also shown. Scale bar 10 μm.

On the other hand, discernable changes in heterochromatin expression were observed after staining for the histone modification H3K9me3 (Figure 3). Comparison of the RFI revealed significant increases in the hypergravity samples in contrast to the 1× *g* CCC and HWC controls (Figure 3A). The cell culture control (CCC; RFI: 1.00 ± 0.009, n = 762) and hardware control (HWC; RFI: 1.00 ± 0.008, n = 1153) exhibited identical RFI averages. In contrast, the 1.8g, 20s samples experienced a rapid 63% increase in H3K9me3 expression (1.63 ± 0.027, n = 359). A further slight increase was observed in the 9g, 20s samples (1.69 ± 0.019, n = 397), followed by a further increase in the 9g, 75s samples (RFI: 1.90 ± 0.02, n = 378), which exhibited statistical significance compared to CCC (*p* = 0.016), HWC (*p* = 0.007) and the 9g, 1h group (*p* = 0.003). Another substantial increase occurred in the 9g, 15m group (RFI: 2.18 ± 0.028, n = 408), reaching the highest H3K9me3 expression among all groups and exhibiting significant differences compared to CCC (*p* = 7.82 × 10^−5^), HWC (*p* = 2.32 × 10^−5^) and 9g, 1h (*p* = 8.35 × 10^−6^), which saw a sharp drop in RFI (0.95 ± 0.0112, n = 864). Significant *p*-values were observed in several groups when compared to both the control and other experimental groups. After one hour of centrifugation at 9× *g*, the H3K9me3 expression was reduced again to 1g control levels of the CCC and HWC control groups (Figure 3C).

Microscopic visualization, as depicted in Figure 3B, further underscores this strong increasing trend observed for human Jurkat T cells exposed to hypergravity, with a significant rise in signal intensity, particularly in the 9g, 75s and 15 min exposure groups. Subsequently, a sharp decline in fluorescence was observed after one hour of centrifugation, with signal intensity levels returning to a state similar to that of the controls. This comprehensive analysis reveals a dynamic response of H3K9me3 histone modification in Jurkat cells under hypergravity conditions.

### 2.4. Quantitative Analysis of Cytoskeletal Changes in Hypergravity

**Figure 4:**
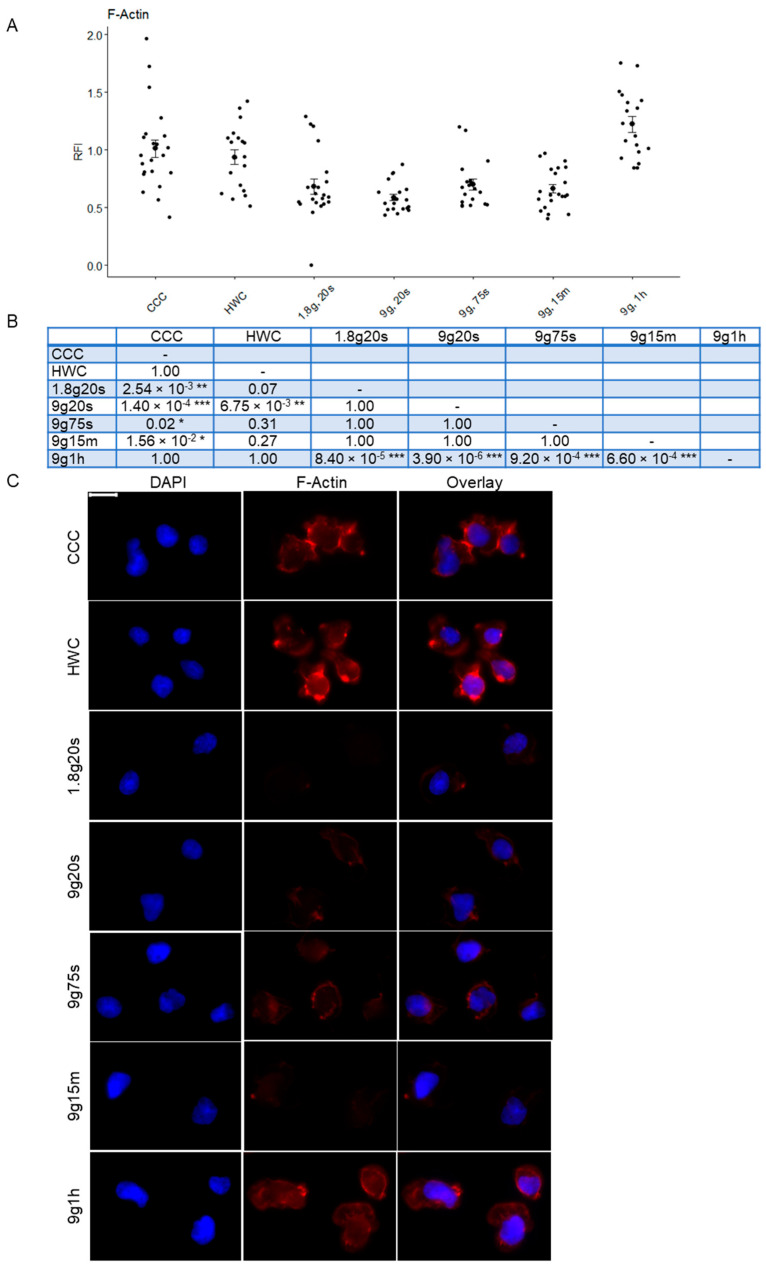
Relative fluorescence intensity (RFI) of F-actin of Jurkat T cells exposed to different hypergravity conditions. (**A**) F-actin was visualized with immunofluorescent staining and subsequently analyzed quantitatively. Each data point represents the mean RFI calculated from all cells within a respective image. Error bars indicate SEM. (**B**) Summary table of *p*-values compared between groups (* *p* < 0.05, ** *p* < 0.01, *** *p* < 0.001). (**C**) Microscopic images of Jurkat T cells exposed to different gravity conditions. F-actin was stained with Phalloidin 647 (red), whereas the nucleus was counterstained with DAPI (blue). Scale bar 10 μm.

Cells were also stained for F-actin to determine possible quantitative changes in cytoskeletal components under different gravity conditions (Figure 4). RFI was measured from Jurkat cells, which revealed significant changes between sample groups (Figure 4A). Similar to the H3K9me3 analysis, the measurement of F-actin staining revealed no significant difference between the RFI of CCC (RFI: 1.01 ± 0.078) and HWC (RFI: 0.94 ± 0.063) groups. This was followed by a subsequent rapid decline in cellular F-actin content for both the 1.8g, 20s (RFI: 0.68 ± 0.065) and 9g, 20s (RFI: 0.59 ± 0.029) groups. A minor increase was observed after 75 s of hypergravity at 9× *g* (RFI: 0.70 ± 0.047), followed by a slight, albeit non-significant, decrease after 15 min (RFI: 0.66 ± 0.038). A significant change was noted in the 9g, 1h samples (RFI: 1.22 ± 0.068), where F-actin fluorescence surpassed that of the control groups CCC and HWC. Several groups exhibited statistically significant *p*-values compared to controls and other experimental groups (Figure 4C). The microscopic examination revealed a reduction in actin filaments under different hypergravity exposure times (Figure 4B). Actin filaments appeared prominently stained around the nuclei in both the cell culture control (CCC) and hardware control (HWC) groups. F-actin signal was dimmed in the hypergravity samples, except for the 1 h exposure group, which showed F-actin signal recovery.

### 2.5. Dynamic Changes of Nuclear Morphology in Hypergravity

**Figure 5:**
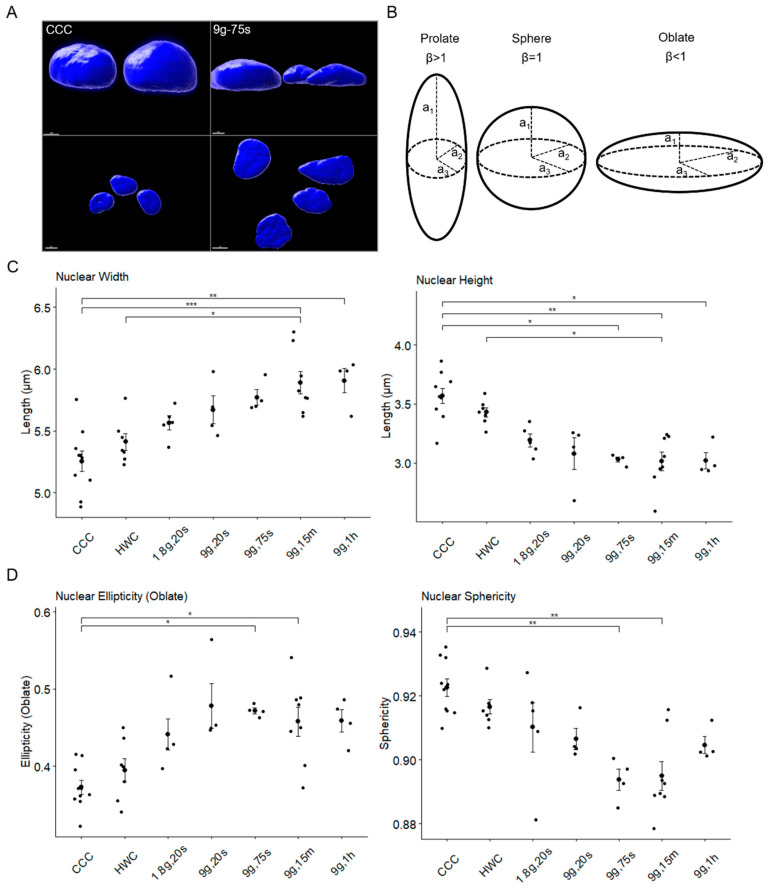
Three-dimensional analysis of nuclear morphology reveals deformations under hypergravity conditions. (**A**) Three-dimensional rendering of surfaces from nuclei in Imaris depicting CCC (**left**) and a 9g, 75s sample (**right**). The top panel shows the visible differences in nuclear height, while the bottom panel illustrates the apparent width of nuclei. Scale bars at 3 μm (**top**) and 5 μm (**bottom**). (**B**) A schematic portraying the vectors measured in spheroids, where a1 resembles the height, a2 and a3 correspond to the widths and β represents the sphericity (β = a1/a2 = a1/a3). (**C**) Plots showing the differences in nuclear width (**left**) and height (**right**) between the different gravitational conditions. (**D**) Plots exhibiting the changes in nuclear ellipticity (**left**) and sphericity (**right**). Single data points and means are shown for each experimental group (* *p* < 0.05, ** *p* < 0.01, *** *p* < 0.001). Error bars indicate SEM.

To investigate possible changes in nuclear morphology, sample groups were also analyzed with confocal microscopy to merge X/Y/Z stacks of Jurkat nuclei into 3D surfaces (Figure 5). Sample sizes for controls and experimental groups were as follows: CCC n = 310 cells; HWC n = 195 cells; 1.8g, 20s n = 122 cells; 9g, 20s n = 134 cells; 9g, 75s n = 111 cells; 9g, 15m n = 116 cells; 9g, 1h n = 177 cells. Nuclear sphericity was evaluated using height and width vectors, where a1 represented nuclear height and a2 corresponded to nuclear width (Figure 5B). Nuclei tended to undergo oblate elliptical transformation under hypergravitational states, with flattened poles and elongated sides (Figure 5A). Consequently, horizontal width (a2) was considered for analysis. Nuclear width exhibited a gradual increase with longer hypergravity exposure (Figure 5C, left). CCC had the lowest values (5.20 µm ± 0.045), while HWC showed a slight non-significant increase in width (5.40 µm ± 0.056). Subsequent groups displayed steady increases, with the 9g, 15m group showing the most significant change in nuclear width (5.82 µm ± 0.079), significantly differing from CCC (*p* = 0.0016) and HWC (*p* = 0.0439). In contrast, nuclear height decreased with longer hypergravity exposure (Figure 5C, right). CCC had the highest nuclear height (3.62 µm ± 0.031), while HWC exhibited a slight non-significant decrease (3.44 µm ± 0.036). A steady decrease was observed in several groups, with the 9g, 75s group displaying the most significant difference (3.03 µm ± 0.051) compared to CCC (*p* = 0.0095).

The analysis of oblate ellipticity and sphericity (Figure 5D) further confirmed that nuclei became progressively more oblate under hypergravity. The CCC group had the lowest oblate ellipticity (0.364 ± 0.006), followed by a slight increase in HWC (0.392 ± 0.008) (Figure 5D, left). The 1.8g, 20s (0.434 ± 0.011) and 9g, 20s (0.460 ± 0.011) groups showed continued increases, with 9g, 75s (0.470 ± 0.011) having statistical significance compared to CCC (*p* = 0.0163). A slight decrease was observed in 9g, 15m (0.449 ± 0.012), which was significant compared to CCC (*p* = 0.0122). After 1 h, there was a minor increase in ellipticity (0.467 ± 0.008). Notably, sphericity decreased with longer hypergravity exposure, mirroring the trend observed in oblate ellipticity (Figure 5D, right). As expected, CCC had the highest sphericity (0.924 ± 0.002), followed by a slight decrease in HWC (0.917 ± 0.002). This decrease continued in both the 1.8g, 20s (0.913 ± 0.004) and 9g, 20s (0.906 ± 0.003) samples. A significant rapid decrease was observed in the 9g, 75s samples (0.896 ± 0.003), with the lowest sphericity compared to CCC (*p* = 0.0095). Sphericity then increased slightly after 15 min of hypergravity (0.898 ± 0.004), maintaining statistical significance against CCC (*p* = 0.0020). Finally, there was a moderate increase in sphericity in the 9g, 1h samples (0.903 ± 0.002). In summary, the longer the nuclei were exposed to hypergravity, the more oblate and less spherical they became, reflecting a flattened shape. However, after 1 h of hypergravity exposure, they began to recover their spherical shape.

### 2.6. Characterization of Histone Modification Spots in Hypergravity

**Figure 6:**
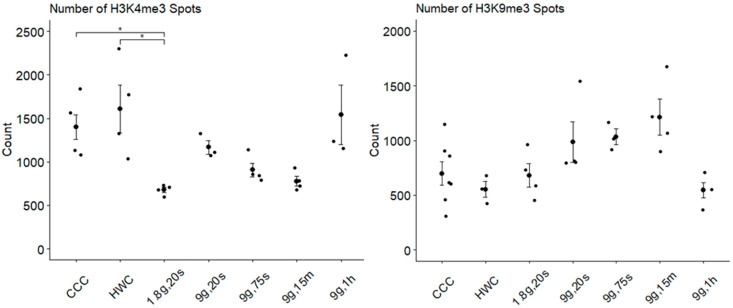
Quantification of histone spots in Jurkat cell nuclei exposed to diverse gravitational conditions. The averaged counts of H3K4me3 (**left**) and H3K9me3 (**right**) histone spots within nuclei for each gravitational condition are displayed. Data points denote mean spot count of a nucleus per image (* *p* < 0.05). Error bars indicate SEM.

In order to characterize the individual histone modification spots and compare gravitational conditions, corresponding fluorescent spots were detected using confocal laser scanning microscopy. The first parameter examined was the average number of histone modification spots inside each nucleus (Figure 6). For H3K4me3, the average number of histone modification spots inside the nuclei was analyzed and compared across various gravitational conditions (Figure 6, left). Cell culture controls (CCC) and hardware controls (HWC) displayed similar numbers of spots within the nuclei. CCC (1404 ± 174, n = 67) had a slightly lower average number of H3K4me3 spots compared to HWC (1617 ± 235, n = 56) but was close to the 9g, 1h sample (1454 ± 205, n = 54). All other experimental groups had fewer H3K4me3 spots. Notably, the 1.8g, 20s group exhibited the fewest histone spots (681 ± 85, n = 61), accounting for roughly 50% of both controls and the longest hypergravity exposure. This group had statistical significance compared to CCC (*p* = 0.035) and HWC (0.033). The number of spots also decreased to varying degrees in the subsequent groups 9g, 20s (1162 ± 178, n = 38); 9g, 75s (897 ± 127, n = 46); and 9g, 15m (778 ± 98, n = 63).

On the other hand, fewer spots were detected for H3K9me3 overall in each group compared to H3K4me3 (Figure 6, right), with an increasing trend starting from CCC (576 ± 68, n = 115) and HWC (533 ± 75, n = 55). The 1.8g, 20s samples (561 ± 87, n = 70) displayed similar spot numbers to CCC but slightly more than HWC. A larger increase was observed in the 9g, 20s (814 ± 135, n = 60) samples, followed by steadier increases in both the 9g, 75s (735 ± 130, n = 59) and 9g, 15m (957 ± 144, n = 69) samples. After this time point, a rapid decrease was observed in the 9g, 1h spots (437 ± 63, n = 68), with values very close to those of the hardware control. There appeared to be a gradual increase in the number of H3K9me3 spots directly correlating with the duration of hypergravity exposure until after 1 h of centrifugation. No statistical significance was observed between any of the experimental groups and the control groups regarding the number of H3K9me3 spots. However, it is worth noting that the closest statistically significant *p*-value was found between the 9g, 15m and 9g, 1h groups (*p* = 0.0671).

### 2.7. Spatial Distribution of Histone Modification Spots in Relation to the Nuclear Periphery in Hypergravity

**Figure 7:**
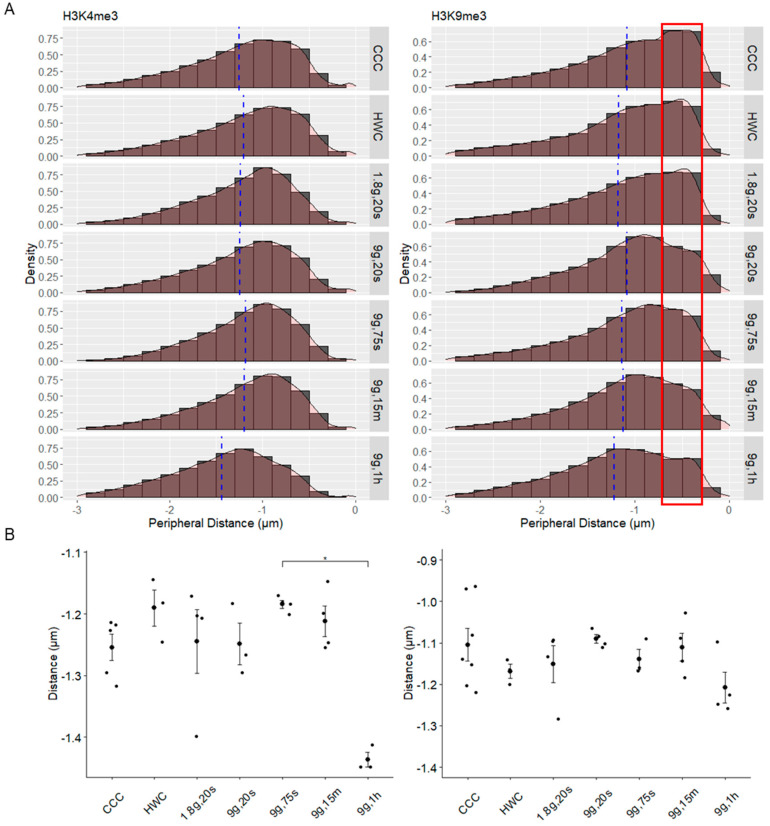
Spatial distribution of histone spots in Jurkat cell nuclei under varying hypergravity conditions. (**A**) Histograms comparing the distribution of spots detected for H3K4me3 (**left**) and H3K9me3 (**right**) and their distance relative to the nuclear periphery. Negative values indicate distances further away from the periphery, i.e., to the center of the nucleus, while “0” represents the nuclear surface. X values are binned at 0.2 μm. Y values are represented as density of the population. Blue dotted lines delineate the means of the groups. The red box denotes the H3K9me3 density peaks within 0.5 μm of the nuclear periphery. (**B**) The means of peripheral distance for each gravity condition were plotted, with individual data points representing the mean distance of all spots in an image (* *p* < 0.05). Error bars indicate SEM.

After demarcating histone modification signals with spots, the distance from these spots to the periphery of the nucleus was also measured (Figure 7). The sample sizes for each group were as follows (n = H3K4me3 spots/H3K9me3 spots): CCC n = 94,046/75,235; HWC n = 73,349/23,834; 1.8g, 20s n = 41,545/3922; 9g, 20s n = 44,174/48,873; 9g, 75s n = 41,257/43,361; 9g, 15m n = 49,034/66,041; 9g, 1h n = 93,510/29,724. The data were plotted on histograms to assess the general distribution of histone modification spots in relation to the periphery of the nuclei (Figure 7A).

In H3K4me3 spots, the histogram data were right-skewed, with negative Pearson skewness coefficient scores for each sample group (Figure 7A, left). While the CCC group had a coefficient score of −0.69, HWC had the most negative score of −0.77, with all other subsequent experimental groups having a more positive score in comparison (1.8g, 20s = −0.72; 9g, 20s = −0.66; 9g, 75s = −0.65; 9g, 15m = −0.73). The 9g, 1h group had the least negative skewness coefficient score of −0.52, with its histogram appearing relatively more normally distributed. This suggests that more H3K4me3 spots have shifted further away from the periphery and concentrated closer to the center of the nucleus after 1 h of centrifugation.

The mean peripheral distance values were also plotted (Figure 7B), and H3K4me3 spot distances appeared relatively consistent among all groups (Figure 7B, left), except for 9g, 75s (−1.18 ± 0.0025)—which had the highest mean—and 9g, 1h (−1.44 ± 0.0019)—which had the lowest values—with statistical significance between these two groups (*p* = 0.0324). HWC (−1.20 ± 0.0022) showed a slight increase compared to CCC (−1.25 ± 0.0019), while both the 1.8g, 20s (−1.24 ± 0.0027) and 9g, 20s (−1.25 ± 0.0027) groups had averages similar to CCC. Meanwhile, the 9g, 15m (−1.20 ± 0.0025) sample’s means were close to that of the HWC group.

The distributions for the H3K9me3 spots appeared even more right-skewed than that of the H3K4me3 distributions, with most spots concentrated closer to the nuclear periphery (Figure 7A, right). Similar to the H3K4me3 histogram, the 9g, 1h sample displayed the least negative skewness compared to the other groups, as indicated by the Pearson’s coefficient scores (CCC = −0.88; HWC = −1.00; 1.8g, 20s = −0.98; 9g, 20s = −0.69; 9g, 75s = −0.95; 9g, 15m = −0.78; 9g, 1h = −0.62). In CCC, HWC and 1.8g, 20s, the peak densities were within 0.5 μm of the nuclear periphery (highlighted in the red box), while the density peaks were observed beyond 0.5 μm but still within 1 μm in all the other experimental groups, indicating that more spots were concentrated slightly away from the periphery but still remained relatively close.

The average distances from the periphery for the H3K9me3 spots appear relatively stable between hypergravitational groups and the controls (Figure 7B, right). CCC (−1.09 ± 0.0023) has a slightly closer distance to the periphery compared to HWC (−1.17 ± 0.0044). The farthest values are seen in the 9g, 1h sample (−1.22 ± 0.0037), even though it is still relatively similar to HWC, and the closest spots are within the 9g, 20s sample (−1.08 ± 0.0026). The remaining samples contained averages lying within this range (1.8g, 20, −1.18 ± 0.0035; 9g, 75s, −1.14 ± 0.0031; 9g, 15m, −1.12 ± 0.0024). Overall, the data suggest that hypergravity exposure did not lead to significant changes in the average distances of the H3K9me3 spots from the nuclear periphery, as the values remained relatively stable across the different experimental conditions.

### 2.8. Dynamic Response of H3K9me3 Histone Modification Spots to Hypergravity

**Figure 8:**
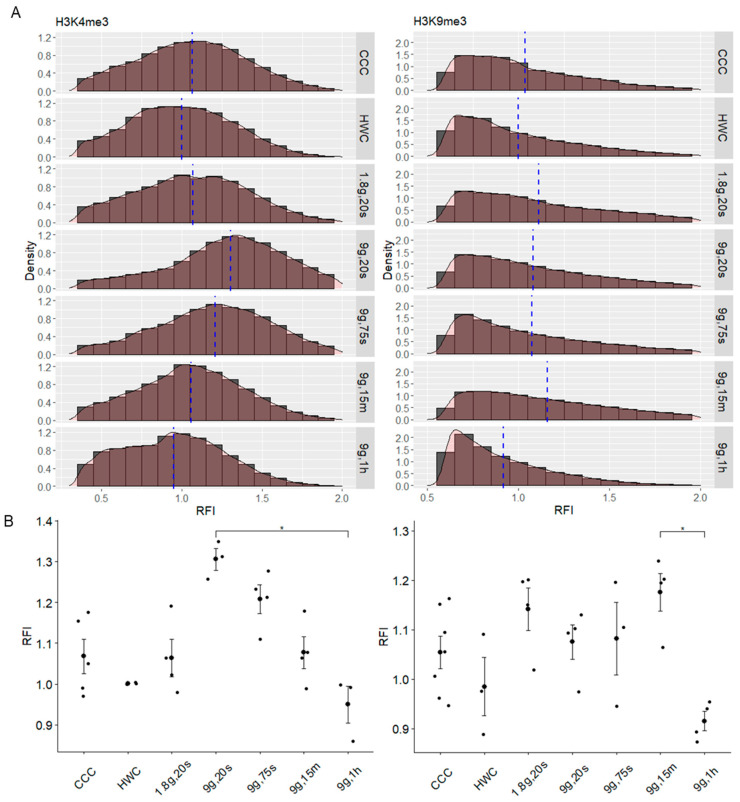
Histone spot fluorescence intensity relative to hypergravity exposure times in Jurkat cell nuclei. (**A**) Histograms comparing the distribution of spots detected for H3K4me3 (**left**) and H3K9me3 (**right**) and their individual RFIs. X values are binned at 0.1 RFI. Y values are represented as density of the population. Blue dotted lines delineate the means of the groups. (**B**) The means of spot RFIs for each gravity condition were plotted, with individual data points representing the mean RFI of all spots in an image (* *p* < 0.05). Error bars indicate SEM.

Histone modification spots were also analyzed for their individual relative fluorescence and compared to different gravitational conditions (Figure 8). The data distribution of RFI values for each spot was measured in histograms for each gravity condition (Figure 8A). In spots labeling H3K4me3 (Figure 8A left), the data appeared relatively normally distributed among each group, which is reflected by the Pearson’s skewness coefficient scores (CCC = 0.25; HWC = 0.21; 1.8g, 20s = 0.66; 9g, 20s = 0.18; 9g 75s = 0.12; 9g 15m = 0.33; 9g, 1h = 0.31). This suggests a relatively consistent distribution of fluorescence for the H3K4me3 spots. However, when examining the mean intensities of the H3K4me3 spot RFIs, values exhibit dynamic changes when experiencing prolonged hypergravity (Figure 8B, left). Fluorescence values are relatively stable between the CCC (1.07 ± 0.0011), HWC (1.00 ± 0.0012) and 1.8g, 20s (1.07 ± 0.0018) groups, whereby then the 9g, 20s (1.30 ± 0.0019) sample exhibits a 30% increase in RFI compared to HWC, followed by a steady decreasing trend among the rest of the experimental group samples (9g, 75s = 1.20 ± 0.0018; 9g, 15m = 1.06 ± 0.0015; 9g, 1h = 0.95 ± 0.0011). Prolonged exposure to hypergravity appears to result in dynamic changes in the mean fluorescence intensity, with short-term hypergravity exposure leading to increased H3K4me3 fluorescence intensity and longer exposure durations, causing a significant reduction in H3K4me3 intensity.

In the H3K9me3 spot RFI, the histogram distributions were more positively-skewed, with values concentrated to the left side of the plots (Figure 8A, right) as evidenced by the Pearson skewness coefficient scores being close to or greater than 1 (CCC = 1.08; HWC = 1.08; 1.8g, 20s = 0.88; 9g, 20s = 0.95; 9g, 75s = 1.08; 9g, 15m = 0.89; 9g, 1h = 1.22). Spots from the longest time point in hypergravity had the highest skewness coefficient score, with more spots concentrated at a lower RFI value. This was also reflected in the means, where the 9g, 1h (RFI: 0.92 ± 0.0016) H3K9me3 spots had the lowest average RFI compared to all other samples (Figure 8B, right). The CCC (RFI: 1.04 ± 0.0012) spots had close RFIs to those from the HWC (RFI: 1.00 ± 0.0022), with a rapid increase from HWC to 1.8g, 20s (RFI: 1.11 ± 0.0020) and a slight decrease in the 9g, 20s (RFI: 1.08 ± 0.0017) sample. The 9g, 75s (RFI: 1.07 ± 0.0019) sample was relatively similar to the 20s time point, but there was another increase after 15 min in hypergravity (9g, 15m RFI: 1.16 ± 0.0016). Due to the rapid drop in RFI between 15 min and 1 h, statistical significance was evident (*p* = 0.019). This significant reduction in fluorescence intensity observed after prolonged hypergravity exposure indicates a dynamic response of the H3K9me3 spots to gravitational conditions.

## 3. Discussion

In this study, we investigated the temporal cellular response of human Jurkat T cells to hypergravity, focusing on changes in histone modifications, cytoskeletal actin structure and nuclear morphology. We investigated the effect of short-term and mid-term exposure to hypergravity between 20 s and up to 60 min. Table 2 provides a summary of the mean data for each parameter measured in the experiment across the different gravity conditions. The overall average values for each gravity condition (columns) are displayed. Green boxes indicate an increase from both control values (grey), while red marks indicate a decrease. This table provides a comprehensive overview of the experiment results, highlighting the variations in different parameters under different gravity conditions.

Cellular responses to mechanical forces pivot on the intricate interplay of integrin-based adhesion with the extracellular matrix (ECM), facilitated by integrin-mediated mechanotransduction. In applied forces, this dynamic relationship undergoes modifications, impacting integrin activation, conformational changes and the formation of the molecular clutch—a linchpin for force transmission between integrins and the actin cytoskeleton, influencing cellular responses to mechanical stimuli [37,38].

However, mechanotransduction is not limited to adherent cells. Mechanical forces exert a significant impact on immune cells, including monocytes/macrophages, neutrophils, natural killer cells, B cells and T cells, encountering both biochemical and mechanical signals during circulation and tissue residence [39]. Specifically, during T cell adhesion to endothelial cells, external shear stress from blood flow is applied, initiating integrin-mediated adhesion strengthened by an increased affinity to ligands and an enhanced connection to the actin cytoskeleton through talin and kindlin recruitment [40,41]. These shear forces are transmitted to the actin cytoskeleton, a key player in mechanosensing and mechanotransduction [42]. T cell activation is force-dependent, with the T cell receptor (TCR) acting as a passive mechanosensor, leading to local actin polymerization upon binding [43]. Mechanical forces in the pN range, particularly around 10 pN, have been shown to trigger cellular reactions, transmitting these forces to the nucleus via the actin cytoskeleton and influencing processes such as gene expression, proliferation, differentiation and cell migration [39,44,45,46,47,48,49,50,51,52]. Despite recent progress in mechanotransduction, further elucidation of the molecular pathways translating mechanical and biochemical signals into nucleus-mediated cellular reactions, including differential gene expression, is needed.

This study revealed that there were no significant changes in the fluorescence of the euchromatin marker, H3K4me3, when comparing hypergravity samples to controls, which is consistent with previous research showing minimal effects of altered gravity conditions on H3K4me3 levels in human mesenchymal stem cells [53]. However, a decrease was observed after a brief exposure to 1.8× *g*, although this response was not observed in the 9g, 20s sample. This result contrasts with the data from the spot detection system, where the mean relative fluorescence intensity (RFI) of individual spots increased in 9× *g* samples but not in 1.8× *g*. The decrease in the number of spots detected in hypergravity samples compared to controls, combined with the higher intensity per spot, might explain the overall stable fluorescence observed in the entire nucleus. This discrepancy could be attributed to the limitations of the Imaris spot detection software, leading to the underestimation of cellular elements [54], which could contribute to these findings.

Although H3K4me3 did not exhibit significant changes in RFI values between the control and hypergravity, other studies have reported alterations in different euchromatin markers, such as H3K4ac, under varied gravitational conditions [55,56,57]. H3K4me3, known to co-localize with acetylated H3K4, shows related distribution patterns with H3K4ac [58,59]. While our previous studies found a decrease in acetylated histones in hypergravity settings [60], the minimal change in H3K4 methylation may be due to a coordinated interaction, possibly through the Set1-containing complex (COMPASS), limiting the accumulation of acetylation marks and attenuating promoter activation [59,61]. These findings suggest that the intricate relationship between H3K4me3 and H3K4ac may be responsible for the differential response seen in acetylated histone levels, where H3K4me3 could regulate H3K4ac levels to maintain genome integrity during stress conditions such as hypergravity [59,62]. This mechanism potentially limits transcriptional initiation and promotes genomic stability in response to hypergravity-induced stress.

Furthermore, we observed a rapid decrease in F-actin fluorescence within 20 s of hypergravity exposure, indicating filament depolymerization and cytoskeletal restructuring. This is consistent with the downregulation of the actin-binding gene LOC643980 in hypergravity samples [21] and in the studies showing heightened sensitivity to immune cell-mediated cytotoxicity after F-actin inhibition [63]. A separate study found similar results in cells exposed to hypergravity, where a decrease in the F-actin to G-actin ratio was observed [63]. Additionally, another study found a concentrated distribution of G-actin around the nucleus in rat cells following a parabolic flight [64], possibly contributing to the mitigation of nuclear actin and the structural maintenance of the nucleus [65]. After 1 h of hypergravity, F-actin fluorescence recovered, mirroring the long-term stability of actin bundles. Studies of macrophages in space revealed a rapid F-actin reduction in microgravity, supporting our findings and the adaptability of the cytoskeleton [25]. This study underscores the dynamic nature of actin in response to altered gravity, although further investigation of G-actin and nuclear actin may be necessary for a comprehensive understanding [66,67].

The nucleus, a central hub for gene expression, becomes a focal point in understanding cellular responses to force transduction, influenced by various molecular players shaped by cytoskeletal forces [68]. Actomyosin and microtubules, which are dynamic elements of the cytoskeleton, work in tandem to control the shape of the nucleus [69]. One study found that the disruption of the intracellular traction force originating from actomyosin causes a shift in the distribution of Lamin A/C, a nucleoskeletal protein, leading to the deformation of the nucleus [69]. This cytoskeleton-induced deformation alters chromatin accessibility, resulting in increased chromatin condensation and influencing gene expression. This mechanotransduction orchestration is further emphasized by the interplay between the actomyosin cytoskeleton, YAP and TAZ transcription factors and the integration of mechanical and biochemical signals [70]. Actin, transitioning between its globular (G-actin) and filamentous (F-actin) forms, plays a pivotal role in mechanotransduction of external forces and regulation of transcription factors imported into the nucleus, influencing gene expression [71]. Nuclear actin (unpolymerized G-actin) has also been found to constantly shuttle between the nucleus and cytoplasm, stabilizing nuclear structure, regulating chromatin dynamics and influencing gene expression [72,73,74]. Mechanical stress or morphological changes further induce cytoskeletal remodeling, and are associated with chromatin condensation and gene silencing [75].

The histone modification H3K9me3 responds sensitively to hypergravity, showing a rapid increase that eventually returns to control levels after 1 h, possibly indicating an adaptation mechanism to maintain genome stability: this experiment showed a rapid 60% increase in H3K9me3 fluorescence within 20 s of exposure to 9× *g* hypergravity, with subsequent increments of about 30% at each examined interval for up to 15 min.

It has been reported that the loss of H3K9 methylation caused heterochromatin disruption, DNA damage and cell death, accompanied by elevated apoptosis-related factors, highlighting the importance of this histone mark in genome stability and DNA damage responses [76]. H3K9me3, working in tandem with HP1, plays a critical role in maintaining heterochromatin and facilitating DNA damage responses [77]. Recent research directly linked H3K9me3 to DNMT1-mediated DNA methylation, reinforcing DNA maintenance and genome stability [78]. Nuclear actin also aids DNA damage repair by relocating heterochromatin to LADs, enabling secure repair of DNA double-strand breaks [79,80]. In the context of hypergravity, the increased H3K9me3 marker and F-actin reorganization likely played a key role in stabilizing the nuclear machinery required for DNA repair and maintenance, thus preventing apoptosis.

Understanding the dynamic modulation of the histone medication H3K9me3 is pivotal, and it involves orchestrated actions by HP1 and the histone methyltransferase (HMT) Suv39h1. These molecular players, known to collaborate under both normal and stress conditions, play a crucial role in preserving genome stability and regulating heterochromatin [81]. Additionally, Lamin A emerges as a regulator, stabilizing both SUV39H1 and HP1, thereby linking heterochromatin remodeling to the LINC complex in lamin-associated domains (LADs). The tethering of heterochromatin is further facilitated by nuclear actin, contributing to the rapid increase and stabilization in H3K9me3 marks on heterochromatin under the influence of hypergravity. This dynamic binding of HP1 to H3K9me3, coupled with Lamin A’s regulatory role, supports the rapid mobilization of nuclear actin, HP1 and Suv39h1 to influence H3K9me3 dynamics in response to mechanical stress [74,82,83].

Furthermore, it is important to note that nuclear morphology is intricately regulated by chromatin compaction, where higher levels of heterochromatin, associated with increased H3K9me3 and H3K27me3, directly correspond to enhanced nuclear rigidity and the preservation of structural integrity, whereas reduced heterochromatin and increased euchromatin lead to an altered nuclear shape [84]. In the context of our experiment, the observed increase in H3K9me3 could represent an adaptive response aimed at mitigating alterations in the nuclear shape induced by hypergravity, thereby aiding in the maintenance of nuclear morphology. This response highlights the cell’s dynamic adaptability to maintain the integrity of its nucleus under changing gravitational conditions.

Consequently, we performed TUNEL analysis and did not detect any significant impact of hypergravity on apoptosis in cells, which is consistent with similar results observed in the transcriptomics of human myelomonocytic U397 cells following parabolic flight and suborbital rocket launch, where no pro-apoptotic or necroptotic effects were observed in both hypergravity and microgravity samples [21]. Interestingly, H3K9me3 changes appear to be linked to distinct mechanistic processes, potentially influenced by force-induced chromatin remodeling mediated by LINC components [85,86]. The experiment revealed maximal H3K9me3 levels after 15 min hypergravity, coinciding with gene expression downregulation observed in previous transcriptomics analyses under similar conditions [24,87]. However, after 1 h of centrifugation, H3K9me3 returned to control values, suggesting a homeostatic adaptation.

Spot distribution of H3K9me3 showed localization near the nuclear periphery, indicative of H3K9me3-enriched heterochromatin. While these spots remained close to the periphery over time, they moved farther from it after 1 h of hypergravity at 9× *g*, implying the potential displacement of this histone modification. The experiment also noted individual H3K9me3 spot fluorescence intensity increasing during hypergravity until it dropped below control levels after 1 h. These findings suggest that H3K9me3 is a highly dynamic modification, regulated by HP1 and HMT Suv39h1, which play roles in maintaining genome stability and heterochromatin regulation under cellular stress [81]. Additionally, the involvement of nuclear actin, HP1 and Suv39h1 in quickly stabilizing H3K9me3 marks on heterochromatin and anchoring them to the nuclear lamins in the LINC complex indicates that LINC proteins in the nuclear envelope contribute to this dynamic chromatin remodeling process [74,82,83,88].

Thus, our main results are as follows: (1) histone modification H3K4me3 showed minimal changes in response to hypergravity, which is consistent with previous research, (2) rapid changes in F-actin fluorescence were observed within seconds of hypergravity exposure, indicating filament depolymerization and cytoskeletal restructuring, whereas after 1 h hypergravity, F-actin fluorescence recovered, reflecting the adaptability of the actin cytoskeleton and (3) histone modification H3K9me3 showed a sensitive response to hypergravity, with a rapid increase within 20 s, which was sustained for up to 15 min and then returned to control levels after 1 h. The dynamic changes of H3K9me3 may play a role in stabilizing nuclear machinery required for DNA repair, thus preventing apoptosis. Our study highlighted the dynamic nature of cellular responses to hypergravity, particularly in terms of histone modifications and cytoskeletal changes. These findings shed light on the adaptation mechanisms that cells employ to maintain genome stability and structural integrity under hypergravity conditions as constantly occurring in the human body during blood cell circulation. Understanding these mechanisms is crucial for various applications, including the study of cellular responses to mechanical forces and the response of the human organism to hypergravity forces induced by high-performance aircrafts and spaceflights.

## 4. Materials and Methods

### 4.1. Cell Culture

Human Jurkat T lymphocytes (ATCC Clone E6-1, TIB-152, Manassas, VA, USA) were cultured in 1640 RPMI (Gibco) medium, supplemented with 10% FBS and 1% Pen/Strep. During an 8 h incubation at 36.5 ± 0.5 °C, the T cells sedimented at the bottom of the cell culture flask to avoid centrifugation. The supernatant was removed, and cell concentration was readjusted to 2.7 × 10^6^ cells/mL. Then, 1 mL cell suspension was aspirated into sterile, pre-warmed (36.5 °C) 1 mL serological pipettes. The pipette tips were sealed with sterile silicone plugs.

### 4.2. Centrifugation Experiment Design

Cell-filled pipettes were placed in an incubator at 36.5 ± 0.5 °C for a duration (T_i_) based on the centrifugation time (T_c_) assigned to each experimental group. This ensured a total incubation time of 15 min (Table 1). Exceptions were made for samples with a centrifugation time of 60 min and cell culture controls (CCC), which deviated from the standard incubation rule. This study comprised twenty-one samples, with three samples per experimental group (Figure 1). Two 1× *g* controls were used. A cell culture control (CCC) and a hardware control (HWC). HWC analyzed cells in the centrifugation hardware without experiencing hypergravity to distinguish which cellular reactions are induced by filling and incubating cells into the hardware. The centrifugation was performed using a custom-made 9× *g* pipette centrifuge (KEK Bad Schmiedeberg, Germany). This unique centrifuge design ensures that the cells are distributed along the entire length of the pipette wall, rather than being collected as pellets at the bottom. Hypergravity samples underwent 9× *g* gravitational force for varying durations (20 s, 75 s, 15 min and 1 h), and an additional group experienced 1.8× *g* for 20 s to simulate parabolic flight conditions. The 9g, 20s group allowed comparison with 1.8g, 20s samples, while the 9g, 75s group simulated a sounding rocket’s launch phase. The 9g, 15m and the 9g, 1h groups served as a longer-term reference to investigate extended effects. With the exception of CCC and hyp60, all samples spent 15 min in the hardware, a duration determined from recent transcriptomic analysis. Adding the 1 h time point aimed to explore any subsequent changes in transcriptomic adaptation. The chosen time intervals facilitated observation of cellular reaction dynamics, comparable to previous transcriptomics experiments and representing initial transient reactions. Following centrifugation, the pipettes were drained into 5 mL sterile, RNAse/DNAse-free plastic tubes containing 1 mL of 8% PFA, and then 1 mL of 4% PFA was aspirated into the pipette, and the pipette was resealed and left to rest at RT for 10 min. Following fixation, the remaining cells were detached by rolling the pipettes for 1 min followed by 5 aspirate/dispense cycles. Samples were then centrifuged (300× *g* for 5 min) and washed with PBS, 3 times. The samples were then stored at 4 °C for further analysis.

### 4.3. Immunocytochemical Staining

Approximately 50,000 fixed human Jurkat T cells were cytospun to each slide for 6 min at a speed of 600 rpm (Shandon Cytospin 3) and then covered in PBS to prevent cells from drying out. All incubation periods were performed in a humidity chamber to prevent evaporation of reagents on slides and to provide protection from light. An aliquot of each sample was analyzed for apoptosis using the Click-iT™ TUNEL Kit (Invitrogen, Waltham, MA, USA) according to the manufacturer’s protocol with DAPI (Enzo, Farmingdale, NY, USA) as the nuclear counterstain. In brief, cells were permeabilized at room temperature with 0.24% TritonX (Sigma-Aldrich, St. Louis, MO, USA) in PBS (Gibco, Waltham, MA, USA) for 20 min followed by two washes in diH_2_O. A DNase solution was applied for 30 min to a positive control. Subsequently, the samples were incubated in TdT Enzyme/Br-dUTP labeling solution at 37 °C for 1 h (negative control received buffer and Br-dUTP only). After washing three times with 3% BSA (Sigma-Aldrich, St. Louis, MO, USA) for 2 min each, slides incubated for 30 min with the Click-iT Reaction solution, protected from light. Following another three washing steps, samples were counterstained with DAPI (2 µg/mL) in PBS for 10 min at room temperature. After a final wash, the slides were embedded on coverslips in mounting medium (Immunomount, DAKO, Santa Clara, CA, USA). In two separate staining procedures, another set of each sample was stained with either anti-H3 tri-methyl lysine 4 (H3K4me3) (ab213224) or lysine 9 (H3K9me3) (ab8898; (Abcam, Cambridge, UK)). In addition to the histone modification of interest, the samples were co-stained for nuclei with DAPI (Enzo) and for F-actin with Phalloidin, Alexa 647 (Invitrogen, Waltham, MA, USA). The cells were permeabilized for 5 min with 0.1% Triton100 at room temperature followed by three 5 min washes with PBS. The samples were then blocked with 3% goat serum (GSA) (Sigma-Aldrich) in PBS for 30 min at room temperature and subsequently incubated with the histone modification primary antibody (H3K4me3 1:100; H3K9me3 1:500) diluted in 0.5% GSA for 1 h at room temperature. After another washing round, slides were incubated with the secondary antibody, Alexa 488 anti-Rabbit (1:100) (Abcam, Cambridge, UK), which was diluted in 0.5% GSA along with Phalloidin (1:40) for 1 h at room temperature protected from light. The samples were again washed with PBS and stained with DAPI (2 µg/mL) diluted in PBS for 10 min in the dark at room temperature. Following a final washing step, the slides were embedded on coverslips in mounting medium (Immunomount, DAKO).

### 4.4. Widefield Microscopy

Samples were imaged using a Leica dMi8 fluorescent inverted microscope with the Leica Application Suite X software version 3.7.4.23463 (Leica Microsystems, Wetzlar, Germany). Three to six images were taken in consecutive order from each slide at 40X (dry, NA 0.95) and another three at 63X (oil, NA 1.4), starting from the border of the coverslip towards the center. All images of a specific epitope were taken with identical parameters for laser intensity, exposure time and gain (Table 3). All signals were detected with the DFT5 Quad filter.

### 4.5. Confocal Microscopy

Representative samples of each group were chosen for confocal laser scanning microscopy. These priority samples were then imaged using a Leica SP5 upright confocal laser scanning microscope and the software “Leica Application Suite X” version 3.7.4.23463 (Leica Microsystems, Wetzlar, Germany). Five to eight images were taken from each slide and the resolution format was chosen as 1024 × 1024 pixels at a dynamic range of 12 bits with a scan speed of 700 Hz. Cells were imaged in X/Y/Z stacks with a pixel size of 45.09 nm × 45.09 nm and stack distance of 0.15 μm, in close accordance with the calculated ideal Nyquist rate for optimal sampling size (Scientific Volume Imaging). A 63X/1.4 oil lens and Hybrid (Power HyD S) detectors were used. Laser intensity, exposure time and gain remained identical for all images of an epitope (Table 4). Alexa Fluor 488 signals (histone modification antibodies) were detected with a laser at 488 nm (emission at 507–602 nm), nuclear staining (DAPI) was detected with a laser at 359 nm (emission at 420–506 nm) and actin (Phalloidin) was detected with a laser at 647 nm (emission at 658–776 nm). Lasers were operated on counting mode and scanned sequentially to avoid channel crosstalk and provide maximum signal yield.

### 4.6. Image Analysis

Images recorded by both widefield and confocal microscopy (LIF files) were analyzed and measured with the image processing software Imaris 9.8.0 (Bitplane, Zurich, Switzerland). TUNEL-stained samples were imaged and analyzed first before moving on to the histone modification staining and analyses. TUNEL-positive cells were counted and summed between each image of a sample, and the apoptotic index (AI) was calculated. Only samples that were TUNEL-negative proceeded to the next immunocytochemical analyses, representing the living cell population.

Images taken with the widefield microscope at 40× consisted of about 50–90 cells per image, examining 8–12 images, resulting in an average of roughly 300–1000 cells analyzed per experimental group. Within each image, the mean relative fluorescent intensity (RFI) was measured for the epitope of each stained cell. The RFI represents a ratio calculated by normalizing the absolute intensity values through the following formula: the mean fluorescence intensity of an experimental sample divided by the mean fluorescence of the appropriate control (in this case, the hardware control). The area of the nuclei was estimated with DAPI staining, and a surface template for the area was defined and seed points were used to identify nuclei. The threshold for absolute intensity was set and the filter “Quality above threshold” was used for this. Mitotic cells, false-positive, non-specific signals and cells’ cut off at the image border were manually excluded. After determination of the cell area of each cell, the RFI of the epitope staining was calculated using the DAPI-deduced area and the signal from the respective epitope staining. The background signal was measured and subtracted, and parameters were adapted for each analyzed image. We then calculated the mean RFI for each image, representing the image-wise average. These image-wise averages were then plotted as the datapoints on scatter plots, representing roughly 8–12 datapoints per group. The mean RFI values reported in the text represent the calculated averages across all cells within the experimental group, encompassing approximately 300–1000 cells per group.

Confocal images underwent Huygens’ deconvolution (Huygens Professional 21.10 SVI), merging up to one hundred single planes for 3D analysis. About 3–5 confocal images with approximately 50–100 cells per sample were analyzed from histone antibody staining experiments. Nucleus-enclosing templates were generated via the “Surface” function using DAPI data, guided by seed points for nucleus identification. The threshold for absolute intensity was set and filtered with “Quality above threshold”. Mitotic cells and cells’ cut off at the image border were manually excluded. Parameters measured from these surfaces were used to analyze nuclear morphology. The “Spot” detector isolated histone markers using Alexa Fluor 488 channel data, with a seed point diameter of ~200 nm. Intensity thresholding (via “Quality above threshold”) was applied after an absolute threshold setting. Nuclear morphology parameters (area, volume, sphericity, oblate ellipticity, height, width) were taken from the nuclear-enclosing surfaces, while histone marker traits (nearest distance, mean RFI, count per nucleus) were automatically measured from the detected spots. The nearest distances between the detected histone spots and the surface of the nuclei were automatically measured and represented the peripheral distance. Background subtraction and uniform parameter settings were maintained during image batch analysis. Mean values of each measured parameter were calculated for each image, representing the image-wise average, which were then plotted as individual data points (n = 3–5) on scatter plots. In the text, overall mean values reported represent the calculated averages across all cells or histone spots within the experimental group.

### 4.7. Statistical Analysis

Image analysis results were exported from Imaris as Excel files (Microsoft Office Professional Plus 2013) and the software R (rStudio v1.4.1717) was used for statistical analysis and the plotting of results. Statistical evaluation of more than three groups were compared by the non-parametric Kruskal–Wallis and Dunn’s multiple comparison test. Values of *p* < 0.05 were considered statistically significant and were adjusted according to the Bonferroni method. Tables showing *p*-values were made to prevent overcrowding in scatter plots. In scatter plots, individual data points represent the image-wise average, determined by calculating the mean channel RFI from each cell within the corresponding image. Error bars indicate the standard error of the mean. The 8-pointed stars, connected to the error bars, signify the collective average of the image means within the experimental group. Results presented in the text are expressed as overall group calculated mean values ± standard error of the mean (SEM). For histograms, the data represent the density or frequency of all data points collected in a group. Blue dotted lines mark the overall mean for that group. Skewness was also measured to analyze the position of the majority of data values in the distribution around the mean value. To investigate the shape of distribution for each gravity condition, Pearson’s skewness index (I) was used.

## Figures and Tables

**Table 1:** List of experimental and control groups used for the hypergravity centrifugation experiment. The gravitational force × *g*, centrifugation time (T_c_) and incubation time (T_i_) for each group are represented here.

ID	Group	Gravitational Force (× *g*)	Centrifuge Time (T_c_)	Incubation Time (T_i_)
CCC	Cell culture control (CCC)	1	0	0
HWC	Hardware control (HWC)	1	0	15 min
1.8g, 20s	Hypergravity (parabolic flight simulation)	1.8	20 s	14 min 40 s
9g, 20s	Hypergravity	9	20 s	14 min 40 s
9g, 75s	Hypergravity (sounding rocket simulation)	9	75 s	13 min 45 s
9g, 15m	Hypergravity	9	15 min	0
9g, 1h	Hypergravity	9	60 min	0

**Table 2:** Summary of mean data for experiment parameters. This table presents a compilation of mean data for various experiment parameters. Each column corresponds to a different gravity condition, with average values displayed. Increases from both control values (grey) are highlighted by green boxes, while decreases are indicated by red marks. The first row provides a summary of relative fluorescence intensity (RFI) values obtained through widefield analysis. Subsequent rows detail parameters concerning nuclear morphology. Rows pertaining to histone modification (HM) spot values encompass information derived from confocal data. These values include the quantification of histone spots, histogram plot skewness and mean values for both H3K4me3 and H3K9me3. Additionally, these rows account for the distance of the histone spots from the nuclear periphery (where more negative values signify proximity to the nuclear center) and their respective RFIs.

Analyses	Epitope/Parameter		CCC	HWC	1.8g20s	9g20s	9g75s	9g15m	9g1h
Widefield Overall RFI	H3K4me3		0.80	1.00	0.74	0.92	0.86	0.97	0.89
	H3K9me3		1.00	1.00	1.63	1.69	1.90	2.18	0.95
	F-Actin		1.01	0.94	0.68	0.59	0.70	0.66	1.22
Nuclear Shape	Width (µm)		5.20	5.40	5.56	5.62	5.77	5.82	5.96
	Height (µm)		3.62	3.44	3.21	3.15	3.03	3.07	2.98
	Oblate		0.36	0.39	0.43	0.46	0.47	0.45	0.46
	Sphericity		0.92	0.92	0.91	0.90	0.89	0.90	0.90
HM Spot Number	H3K4me3		1404	1617	681	1162	897	778	1454
	H3K9me3		576	533	561	814	735	957	437
HM Spot Peripheral Distance	H3K4me3	Skew	−0.69	−0.77	−0.72	−0.66	−0.65	−0.73	−0.52
		Mean (µm)	−1.25	−1.20	−1.24	−1.25	−1.18	−1.20	−1.44
	H3K9me3	Skew	−0.88	−1.00	−0.98	−0.69	−0.95	−0.78	−0.62
		Mean (µm)	−1.09	−1.17	−1.18	−1.08	−1.14	−1.12	−1.22
HM Spot RFI	H3K4me3	Skew	0.25	0.21	0.66	0.18	0.12	0.33	0.31
		Mean	1.07	1.00	1.07	1.30	1.20	1.06	0.95
	H3K9me3	Skew	1.08	1.08	0.88	0.95	1.08	0.89	1.22
		Mean	1.04	1.00	1.11	1.08	1.07	1.16	0.92

**Table 3:** Widefield microscopy imaging parameters. Samples were imaged on a widefield fluorescence microscope with specific epitopes having identical parameters for excitation and emission wavelengths, exposure time, laser power and gain.

Epitope	Excitation (nm)	Emission (nm)	Exposure Time (ms)	Laser Power	Gain
DAPI	390	460–480	100	60%	low noise & high well
H3K4me3/H3K9me3 (Alexa 488)	475	535–570	200	60%	low noise & high well
Phalloidin (Alexa 647)	635	>695	300	60%	low noise & high well
TUNEL	635	>695	200	40%	low noise & high well

**Table 4:** Confocal microscopy imaging parameters. Samples were imaged on a confocal laser scanning fluorescence microscope with specific epitopes having identical parameters for excitation and emission wavelengths, exposure time, laser power and gain.

Epitope	Excitation (nm)	Emission (nm)	Exposure Time (ms)	Laser Power	Gain
DAPI	405	425–506	100	30%	2
H3K4me3/H3K9me3 (Alexa 488)	488	506–620	200	50%	2
Phalloidin (Alexa 647)	647	650–776	300	50%	2

## Data Availability

The data presented in this study are available on request from the corresponding author. The data are not publicly available in a public repository due to the data volume and format (imaging data).

## Supplementary Materials

The supporting information can be downloaded at: https://www.mdpi.com/article/10.3390/ijms242417232/s1.

## Abbreviations

LINC	Linker of nucleoskeleton to cytoskeleton
RFI	Relative fluorescence intensity
SEM	Standard error of mean
CCC	Cell culture control
HWC	Hardware control
NC	Negative control
PC	Positive control
H3K4me3	Histone 3 lysine 4 trimethylation
H3K9me3	Histone 3 lysine 9 trimethylation
H3K4ac	Histone 3 lysine 4 acetylation
H3K27me3	Histone 3 lysine 27 trimethylation
HM	Histone modification
TUNEL	Terminal deoxynucleotidyl transferase dUTP nick end labeling
COMPASS	Complex Proteins Associated with Set1
HMT	Histone methyltransferase
LAD	Lamin associated domain
