# Supplementary material for: Increased H3K9me3 and F-Actin Reorganization in the Rapid Adaptive Response to Hypergravity in Human T Lymphocytes
Source: Int J Mol Sci. 2023 Dec 7;24(24):17232. doi: 10.3390/ijms242417232 (PMC10743231; doi:10.3390/ijms242417232)
Supplement: Supplementary file 1 [file ijms-24-17232-s001.zip › ijms-2721718-supplementary.pptx]

## Slide 1
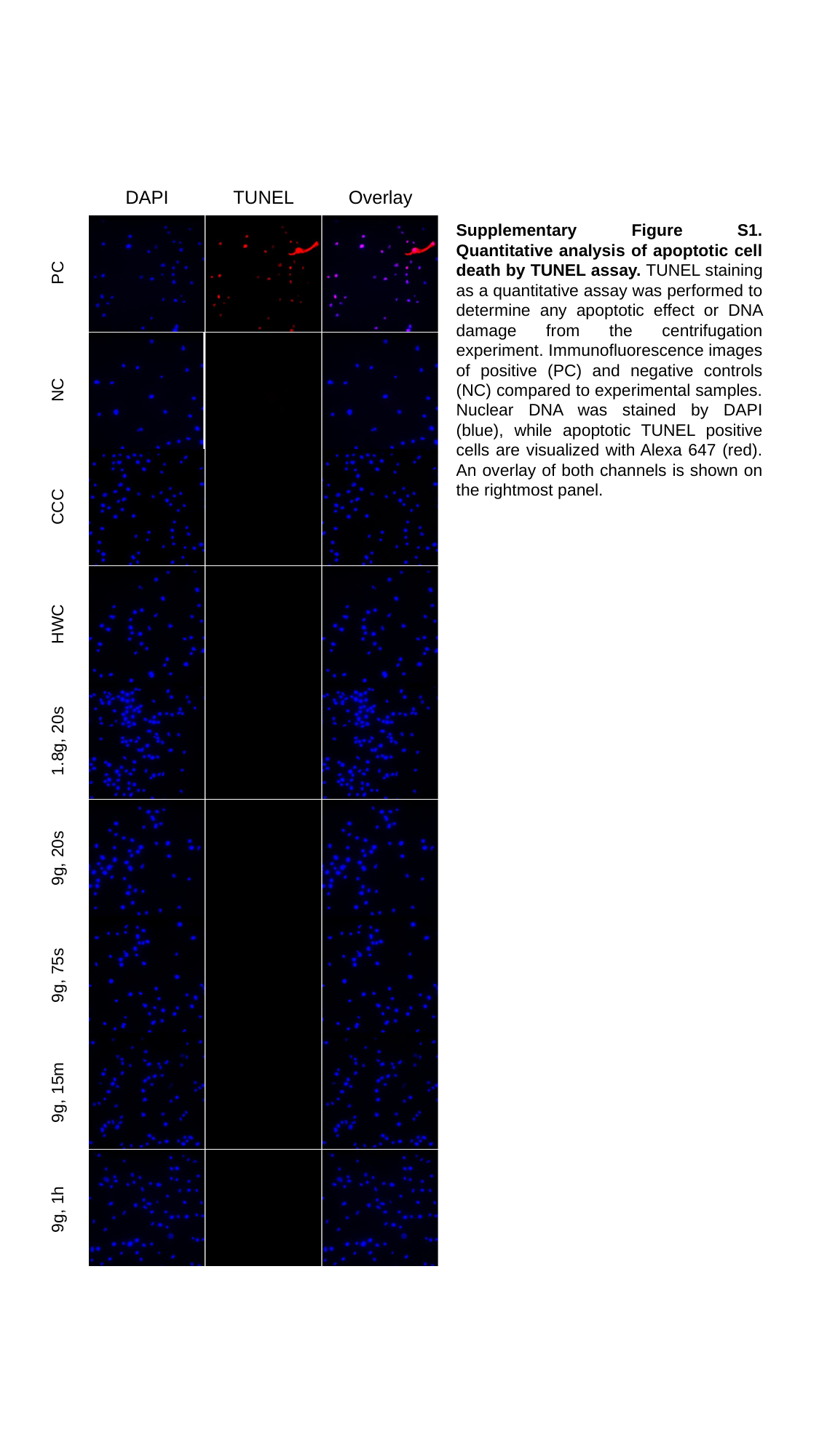

| PC |
| --- |
| NC |
| CCC |
| HWC |
| 1.8g, 20s |
| 9g, 20s |
| 9g, 75s |
| 9g, 15m |
| 9g, 1h |
Supplementary Figure S1. Quantitative analysis of apoptotic cell death by TUNEL assay. TUNEL staining as a quantitative assay was performed to determine any apoptotic effect or DNA damage from the centrifugation experiment. Immunofluorescence images of positive (PC) and negative controls (NC) compared to experimental samples. Nuclear DNA was stained by DAPI (blue), while apoptotic TUNEL positive cells are visualized with Alexa 647 (red). An overlay of both channels is shown on the rightmost panel.
